# Characterization of pathological features and immune microenvironment in hepatic tuberculosis and pulmonary tuberculosis

**DOI:** 10.3389/fcimb.2024.1418225

**Published:** 2024-09-30

**Authors:** Qiang Niu, Runrui Wu, Ke Pan, Xinlan Ge, Wen Chen, Rong Liu

**Affiliations:** ^1^ Faculty of Hepato-Pancreato-Biliary Surgery, The First Medical Center of Chinese PLA General Hospital, Beijing, China; ^2^ Department of Pathology, The Eighth Medical Center of Chinese PLA General Hospital, Beijing, China

**Keywords:** hepatic tuberculosis, pathology, granulomas, immune microenvironment, macrophage

## Abstract

Hepatic tuberculosis (HTB) is rare extrapulmonary tuberculosis that is clinically similar to liver malignancy, making it difficult for correct diagnoses. Pathology is the gold standard for tuberculosis diagnosis. However, there are few reports on the pathological features of HTB. A total of 32 HTB cases were considered and the differences in pathological features and drug resistance were analyzed and compared with those for pulmonary tuberculosis (PTB). Enhanced CT scans showed ring-shaped delayed enhancement during the arterial, venous, and delayed phases. Most HTB cases were single lesions, with the highest incidence in the right lobe, and the average lesion volume was smaller than that of PTB. The frequency of granuloma in pathological changes, the overall share of the lesion area in the HTB group, and the number of foxp3^+^ cells were significantly higher than in the PTB group. However, no statistically significant differences were observed between the two groups’ other pathological features and immune cell numbers. The immune microenvironment of the normal tissues surrounding the lesion was further analyzed. The findings showed that the number of macrophages and foxp3^+^ cells in the HTB group was significantly higher than in the PTB group. No significant difference in drug resistance was detected between the HTB and PTB groups. In conclusion, there are substantial differences in the characterization of pathological feature and immune microenvironment between HTB and PTB. The frequency of granuloma and subsequent overall share of the lesion area was significantly higher in HTB compared to PTB.

## 1 Introduction

*Mycobacterium tuberculosis* (MTB) usually targets the lungs though it can also infect any other organ to cause extrapulmonary infections (Howard and Khader, 2020; Kanabalan et al., 2021). Hepatic tuberculosis (HTB) is a rare manifestation of extrapulmonary tuberculosis, usually caused by the dissemination of the TB bacilli through the blood, lymph, or invasion of adjacent organ tuberculosis foci (Bova et al., 2023). Epidemiological studies have shown that immunocompromised patients have a significantly higher risk of developing hepatic tuberculosis (Choudhury et al., 2021). The incidence rate of tuberculosis in HIV-infected patients is 20 times higher than that in non-tuberculosis populations. When the CD4^+^ T-cell count is below 200/μL, the incidence rate of HTB and other types of extrapulmonary tuberculosis markedly increases (Picon et al., 2007; Longo et al., 2022). Hepatic tuberculosis lacks typical clinical manifestations, with the most common signs and symptoms being liver enlargement, fever, abdominal pain, and weight loss. A few patients do not present with any symptoms (Giri, 2022). Abnormal liver function may occur, accompanied by elevated levels of alkaline phosphatase and γ-glutamyltransferase. In more than half of the HTB patients, MTB would have infected the liver and damaged its cells long before diagnosis. Usually, the incurred liver damage is irreversible (Liu et al., 2022). Therefore, early diagnosis and timely personalized treatment are key factors in improving the prognosis of hepatic tuberculosis.

Currently, the diagnosis of tuberculosis is mainly based on the patient’s medical history, clinical symptoms, signs, laboratory tests, and imaging examinations. Abdominal-enhanced CT is the best imaging examination for diagnosing hepatic tuberculosis, represented by one or more low-density nodules (Jha et al., 2023). However, imaging examinations sometimes make it difficult to distinguish hepatic tuberculosis from liver abscesses and liver malignancies such as hepatocellular carcinoma, intrahepatic cholangiocarcinoma, and hilar cholangiocarcinoma (Wilhelmi, 2021). Pathology is the gold standard for diagnosing tuberculosis, with H&E staining, acid-fast staining, and molecular detection being among the commonly used tests. The typical pathological feature of tuberculosis is chronic granulomatous inflammation accompanied by caseous necrosis. In terms of organizational characteristics, the liver is a substantial organ with relatively denser tissue and no internal cavities such as those found in the lungs. When the amount of necrotic materials exceeds a certain threshold, following the proliferation of *Mycobacterium tuberculosis* in the liver, macrophages may not be sufficient to eliminate them, and meanwhile, chemotherapy drugs cannot enter the interior to kill the mycobacteria, causing significantly compromised liver tissue healing. In addition, the liver is a complex immune organ that has both immunomodulatory and phagocytic effects. Foxp3 is a transcription factor that directly regulates the expansion and function of T-cells, a role that markedly contributes to maintaining immune tolerance in the liver. The abundant macrophages in the liver can recognize and clear MTB (Ansari et al., 2023). However, the liver also has certain immune regulatory functions, which may interfere with the timely clearance of MTB. The differences in immunity and organizational structure may lead to significant differences between the pathological features of HTB and those of pulmonary tuberculosis (PTB). Currently, few reports that focus on the pathological differences between pulmonary and hepatic tuberculosis are available.

Anti-tuberculosis drugs should be used throughout the entire treatment process and the principles of “early, regular, full process, combination, and moderate” should be strictly followed (Abulfathi et al., 2019). Patients with mild hepatic tuberculosis can be treated using anti-tuberculosis drugs. Surgical treatment should be considered for patients with severe conditions, extensive fibrous wrapping, tuberculous nodules, or massive calcific abscesses (Tonesi, 2021). Some of the common surgical methods are partial hepatectomy and hepatectomy, which reduce the compression of the lesion tissue on the liver hilum and improve related symptoms. If the patient cannot undergo surgical treatment, interventional strategies such as hepatic artery infusion chemotherapy, or hepatic artery chemotherapy embolization, can also be considered (Ansari et al., 2023). Regardless of the treatment method, anti-tuberculosis drugs remain necessary to keep *Mycobacterium tuberculosis* in a resting state. However, the liver toxicity of the drugs should be minimized as much as possible to maintain liver function (Azzaza et al., 2020). Therefore, personalized selection of anti-tuberculosis drugs is crucial when treating hepatic tuberculosis. Thus, understanding the drug resistance characteristics of hepatic tuberculosis has important clinical value. In this article, we compared the differences in the pathological characteristics and immune microenvironment associated with HTB and PTB. This comparison improves the pathological diagnosis rate of atypical HTB, while also providing important references for clinical treatment and basic research.

## 2 Materials and methods

### 2.1 Sample collection

Patients admitted to the Eighth Medical Center of the Chinese PLA General Hospital from January 2012 to March 2024 were included in this retrospective study. The inclusion criteria were as follows: age > 18 years old; liver surgery or puncture; pathological examination; clinical and pathological diagnosis of MTB infection; no prior anti-tuberculosis drug treatment before diagnosis. The exclusion criteria were as follows: malignant tumors; bronchial tuberculosis alone; severe fungal and bacterial infections; malignant hematologic diseases; severe trauma and surgical history. The patients who met the criteria were assigned to the HTB group (n = 32) and PTB group (n = 32). Based on clinical and imaging examinations, no evidence of tuberculosis infection in the liver was found in the PTB group.

Puncture or surgical specimens were immediately fixed in 10% formalin. After 6 hours, the samples were dehydrated by gradient alcohol and embedded to make wax blocks, which can be stored at room temperature for several years. This study was approved by the Ethics Committee of the Chinese PLA General Hospital and was performed as per the principles of the Declaration of Helsinki.

### 2.2 H&E staining

A 3 µm thick slice was cut from the wax block and baked at 72 degrees for 30 minutes. Dewaxing in xylene was done two times, each spanning 10 minutes. For rehydration, 100%, 90%, and 80% gradient ethanol was used for 5 minutes each, before the hematoxylin dye (Sigma) was applied for 2 minutes. Then, 1% hydrochloric acid in 70% ethanol was used to differentiate between nuclear and non-nuclear structures. The ammonia solution was used to turn the red hematoxylin to blue. The eosin dye (Sigma) was applied for 5 seconds, followed by 75%, 85%, 95%, and 100% ethanol gradient dehydration, and clearing by xylene. The sheet was finally sealed with neutral resin.

### 2.3 Acid-fast staining

A 3 µm thick slice was cut from the wax block and was baked at 72 degrees for 30 minutes. This was followed by dewaxing in xylene for 10 minutes, twice. Rehydration was done using 100%, 90%, and 80% gradient ethanol for 5 minutes each time. Carbolic acid redness dye solution (2-3 drops) was added at room temperature for 2-3 hours, before decolorizing with 1% hydrochloric acid alcohol to light pink. This was followed by 1% hydrochloric acid ethanol differentiation, bluing using ammonia, and application of the eosin dye for 5 seconds. After that, 75%, 85%, 95%, and 100% ethanol gradient dehydration and clearing by xylene followed, before sealing with neutral resin.

### 2.4 Mycobacterium strain identification

**Figure 1:**
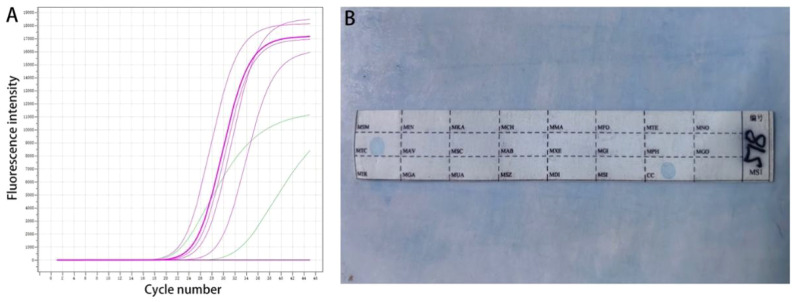
Molecular detection. **(A)** Real-time fluorescence quantitative PCR; **(B)** The sequence of the detection site on the membrane strip.

About 8-10 pieces of tissue, with a thickness of 5-10 µm, were cut from the wax block and placed in a 1.5 mL centrifuge tube. After dewaxing, cracking, digestion, and DNA extraction followed. The PCR tube was removed from the Mycobacterium species identification gene test kit (Shenzhen Yanan Biotechnology Co., Ltd) and 4 μL of sample DNA were added. After amplification, the membrane strip and amplified product were placed together in a test tube containing 5-6 mL of Solution A. The test tube was then heated in a boiling water bath for 10 minutes and hybridized at 59°C for 1.5 hours in a hybridization oven. The membrane strip was removed and placed in a test tube that contained 40 mL of Solution B. This was washed in the hybridization oven for 15 minutes, before incubating in solution A containing the peroxide enzyme for 30 minutes. Finally, the membrane strip was placed in the developing solution and kept away from light for 10 minutes. The reaction was then stopped using deionized water. As shown in **Figure 1**, blue spots on the membrane indicated successful detection of the target site (Hardman et al., 1996; He et al., 2022).

### 2.5 Immunohistochemical staining

After paraffin sectioning, the slides were dewaxed in xylene for 30 minutes, treated with 3% hydrogen peroxide, and then subjected to antigen blocking using a sheep serum working solution (ZSGB-BIO). The primary antibody (All purchased from Abcam) was added before incubating for 16 hours in a 4°C refrigerator. After washing three times with PBS, the biotinylated secondary antibody (ZSGB-BIO) was added and incubation was done for 4 hours at 37°C. PBS was used to wash three times before adding the alkaline phosphatase-labeled streptavidin working solution (ZSGB-BIO). After washing for another three times, immunohistochemical staining was carried out, followed by gently rinsing with tap water until the appropriate color depth was displayed. The slide was then placed in a hematoxylin solution for counterstaining. The slide was dehydrated, transparentized, sealed, and finally observed under a microscope.

### 2.6 Drug resistance detection

Sample DNA (4 μL) was added to the PCR tubes from the MTB drug resistance mutation gene detection kit (Shenzhen Yanan Biotechnology Co., Ltd). After DNA amplification, the membrane strip and amplification product were placed in a tube containing 5-6 mL of the AT solution, heated in a boiling water bath for 10 minutes, and then hybridized at 59°C for 1.5 hours. After the washing and incubation procedure, the test strip was placed in the color development solution for 10 minutes. Deionized water was used to stop the reaction. The blue spots on the membrane strip indicated the detected sites (Rao et al., 2016; Liang et al., 2017). The definition of drug resistance types was based on our previous paper (Wu et al., 2023; Li et al., 2024).

### 2.7 Statistical analysis

The data was statistically analyzed using the GraphPad 5.0 software. The comparisons between groups were performed using chi-square or t-tests. A *P* value of < 0.05 was considered statistically significant.

## 3 Results

### 3.1 Clinical data

**Figure 2:**
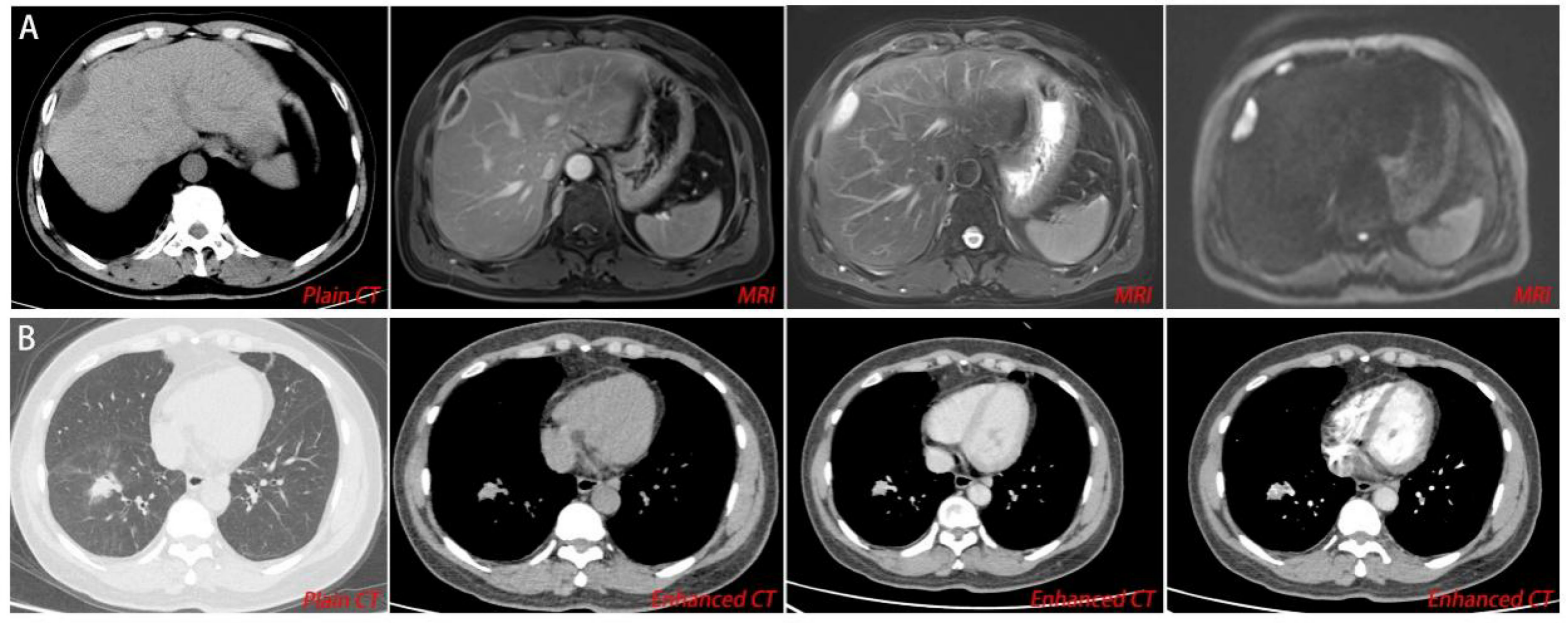
Imaging examination. **(A)** For hepatic tuberculosis, CT and MRI showed ring-shaped delayed enhancement during the arterial, venous, and delayed phases. **(B)** For pulmonary tuberculosis, enhanced CT scans showed uneven enhancement, moderate blood flow perfusion, adjacent bronchial stenosis, compression displacement, and vascular shadows passing through.

The PTB group had 32 patients, with 18 being males and 14 were females. The age range was 18-65 years old, with 41 years being the mean. The average lesion size was 25.84 mm^3^. The HTB group had 32 patients, including 16 males and 16 females. The average age in this group was 38 years old while the mean lesion size was 10.06 mm^3^ (**Table 1**). In 13 patients with hepatic tuberculosis, a clear history of tuberculosis in other parts was noted as follows: 11 cases of pulmonary tuberculosis; 1 case of peritoneal tuberculosis; 1 case of abdominal wall tuberculosis. The remaining 19 cases were more likely to be primary hepatic tuberculosis. The CT and MRI manifestation of hepatic tuberculosis was a patchy mixed signal shadow, with equal length T1 and T2 signals as the main features, mixed with long T1 signal shadows, limited DWI diffusion, and low ADC value. Enhanced scanning showed circular delayed enhancement in arterial, venous, and delayed phases. The CT manifestation of pulmonary tuberculosis was irregular nodular shadows, with blurred edges, uneven enhancement on enhanced scans, moderate blood flow perfusion, adjacent bronchial stenosis, compression displacement, and vascular shadows passing through (**Figure 2**).

**Table 1:** Comparison of clinical data and important pathological features.

	HTB (n = 32)	PTB (n = 32)	*P* value
Gender (male)	16	18	0.803
Age (year)	38	41	0.513
TB Type (MTB)	100% (n = 32)	100% (n = 32)	/
Lesion size (mm^3^)	10.06	25.84	0.003
Methods			
Acid-fast (+)	46.88% (n = 15)	40.63% (n = 13)	0.801
PCR (+)	100% (n = 32)	100% (n = 32)	/
Pathology			
Granuloma	84.38% (n = 27)	53.13% (n = 17)	0.007
Caseous necrosis	40.63% (n = 13)	31.25% (n = 10)	0.435
Inflammatory necrosis	25% (n = 8)	18.75% (n = 6)	0.545
Abscess	6.25% (n = 2)	9.38% (n = 3)	0.641
Exudation	18.75% (n = 6)	28.13% (n = 9)	0.376
Acute inflammation	28.13% (n = 9)	37.50% (n = 12)	0.425
Granulation tissue	21.88% (n = 7)	31.25% (n = 10)	0.396
Fibrous hyperplasia	18.75% (n = 6)	12.50% (n = 4)	0.491

### 3.2 Lesion sites

In the HTB group, 22 cases were single lesions, distributed as follows: under the liver capsule (5 cases); outside the liver capsule (4 cases); left lobe (1 case); and right lobe (12 cases). Ten of the cases in the HTB group were multiple lesions, distributed as follows: under the liver capsule (4 cases); outside the liver capsule (1 case); left lobe (2 cases); and right lobe (3 cases). In the PTB group, there were 13 cases of lesions in the left lobe of the lung and 19 cases in the right lobe.

### 3.3 Pathological features

**Figure 3:**
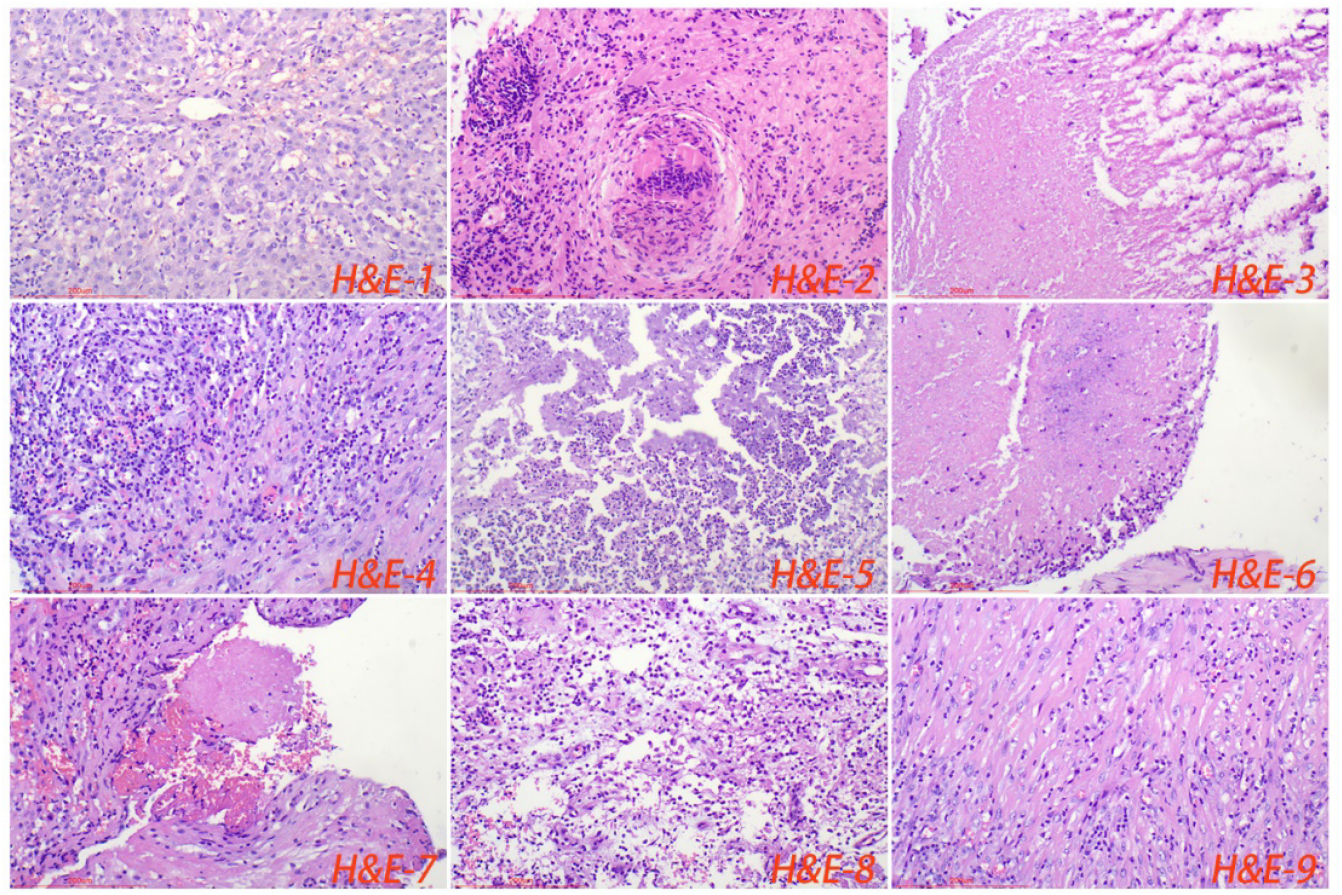
Normal liver tissue surrounding the lesion, with closely connected cells and no obvious lacunae (H&E-1). The typical pathological manifestations of hepatic tuberculosis were chronic granulomatous inflammation (H&E-2) with caseous necrosis (H&E-3). H&E staining showed acute inflammation (H&E-4), abscess (H&E-5), inflammatory necrosis (H&E-6), exudation (H&E-7), granulation tissue (H&E-8), and fibrous tissue hyperplasia (H&E-9). Image magnification: H&E staining (200×).

**Figure 4:**
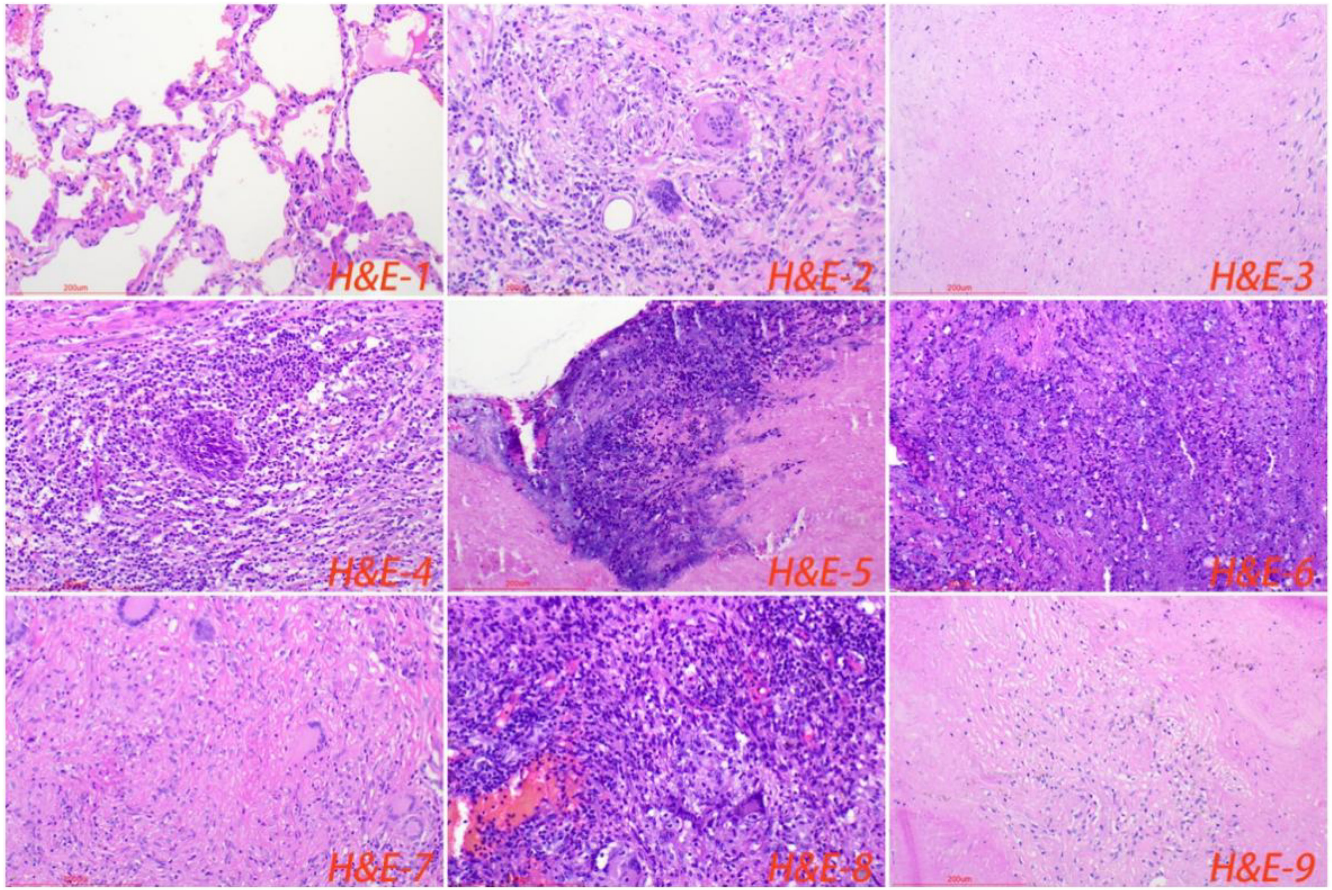
Normal lung tissue surrounding the lesion, with loose cell connections and visible alveolar cavities (H&E-1). The typical pathological manifestations of pulmonary tuberculosis were chronic granulomatous inflammation (H&E-2) with caseous necrosis (H&E-3). H&E staining showed acute inflammation (H&E-4), abscess (H&E-5), inflammatory necrosis (H&E-6), exudation (H&E-7), granulation tissue (H&E-8), and fibrous tissue hyperplasia (H&E-9). Image magnification: H&E staining (200×).

All cases of HTB and PTB were positive for molecular detection. The positive rate of acid-fast staining in the HTB group was 46.88%, while it was 40.63% in the PTB group. There was no statistical difference between the two groups. Among patients with HTB, the frequency of granuloma, caseous necrosis, inflammatory necrosis, abscess, exudation, acute inflammation, granulation tissue, and fibrous tissue hyperplasia were 84.38% (n = 27), 40.63% (n = 13), 25% (n = 8), 6.25% (n = 2), 18.75% (n = 6), 28.13% (n = 9), 21.88% (n = 7), and 18.75% (n = 6), respectively. For patients with PTB, the frequency of granuloma, caseous necrosis, inflammatory necrosis, abscess, exudation, acute inflammation, granulation tissue, and fibrous tissue hyperplasia were 53.13% (n = 17), 31.25% (n = 10), 18.75% (n = 6), 9.38% (n = 3), 28.13% (n = 9), 37.5% (n = 12), 31.25% (n = 10), and 12.50% (n = 4), respectively. Statistical analysis showed that the frequency of granuloma in the HTB group was significantly higher than that in the PTB group. No statistical difference was observed in other pathological features between the two groups. The share of each pathological feature in the entire lesion area was further analyzed and the results showed that granuloma was significantly more prevalent in the HTB group than in the PTB group. However, there was no statistical difference in the prevalence of caseous necrosis between the two groups (**Figures 3**, **4**; **Table 1**).

### 3.4 Immune microenvironment of the lesion regions

**Figure 5:**
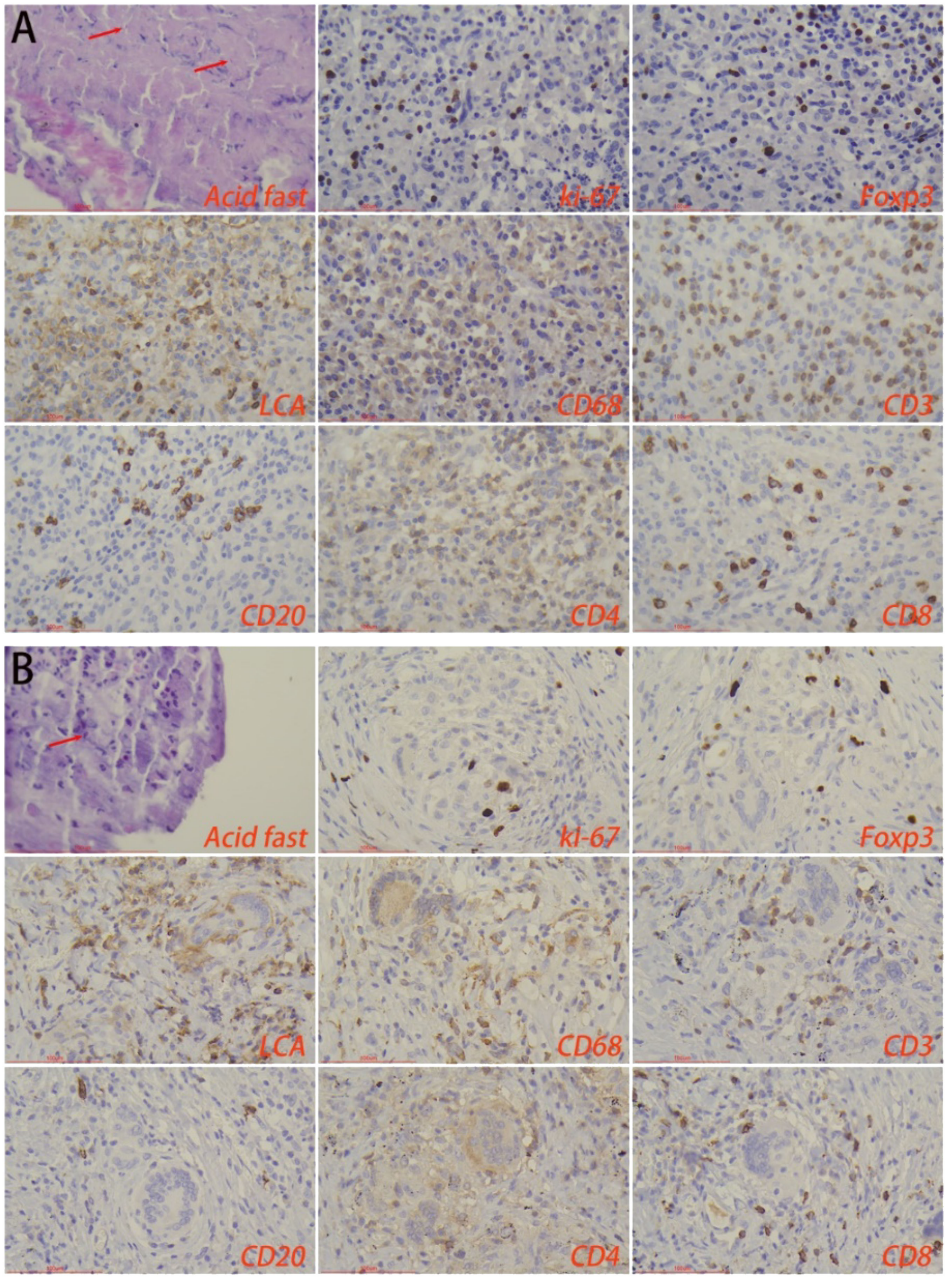
Immune microenvironment of lesion regions in **(A)** hepatic tuberculosis and **(B)** pulmonary tuberculosis. Acid-fast staining showed the presence of *Mycobacterium tuberculosis*. Immunohistochemical staining was used to label proliferation rate (ki-67^+^), immunosuppressive cells (foxp3^+^), lymphocytes (LCA^+^), macrophages (CD68^+^), T-cells (CD3^+^), B-cells (CD20^+^), CD4^+^ T-cells, and CD8^+^ T-cells. Image magnification: Immunohistochemical staining (200×), acid-fast staining (400×).

Immunohistochemical staining showed that there was no statistical difference in the number of macrophages, lymphocytes, T-cells, B-cells, CD4^+^ T-cells, and CD8^+^ T-cells in the lesion area between the HTB and PTB groups. Also, no statistical difference was noted in the proliferation rate of immune cells. Foxp3 is a major marker for inhibitory immune cells (Georgiev et al., 2019; Wang et al., 2023). The results from this study showed that the number of foxp3^+^ cells in the lesion area of the HTB group was significantly higher than in the PTB group (**Figure 5**; **Table 2**).

**Table 2:** Comparison of the immune microenvironment in tuberculosis foci and surrounding normal tissues.

Cell type	Hepatic tuberculosis	Pulmonary tuberculosis	*P* value
Tuberculosis foci			
Macrophage	48.00 ± 4.93	51.33 ± 4.51	0.846
Lymphocyte	37.50 ± 4.51	33.67 ± 8.70	0.704
T-cells	30.17 ± 6.04	17.66 ± 5.12	0.146
B-cells	9.65 ± 2.92	17.00 ± 4.72	0.216
CD4^+^ T-cells	22.13 ± 5.45	13.52 ± 3.42	0.223
CD8^+^ T-cells	18.14 ± 7.11	4.33 ± 2.17	0.092
Foxp3^+^cell	13.51 ± 1.49	5.50 ± 1.12	0.003
Surrounding normal tissue			
Macrophage	14.23 ± 1.63	8.35 ± 1.45	0.020
Lymphocyte	27.00 ± 3.15	21.17 ± 2.93	0.205
T-cells	20.00 ± 2.58	14.83 ± 1.74	0.128
B-cells	7.06 ± 2.67	9.17 ± 1.78	0.469
CD4^+^ T-cells	8.83 ± 2.27	8.50 ± 3.18	0.934
CD8^+^ T-cells	11.50 ± 2.05	6.67 ± 2.43	0.159
Foxp3^+^cell	4.68 ± 0.88	1.36 ± 0.42	0.007

### 3.5 Immune microenvironment of normal tissues surrounding the lesions

**Figure 6:**
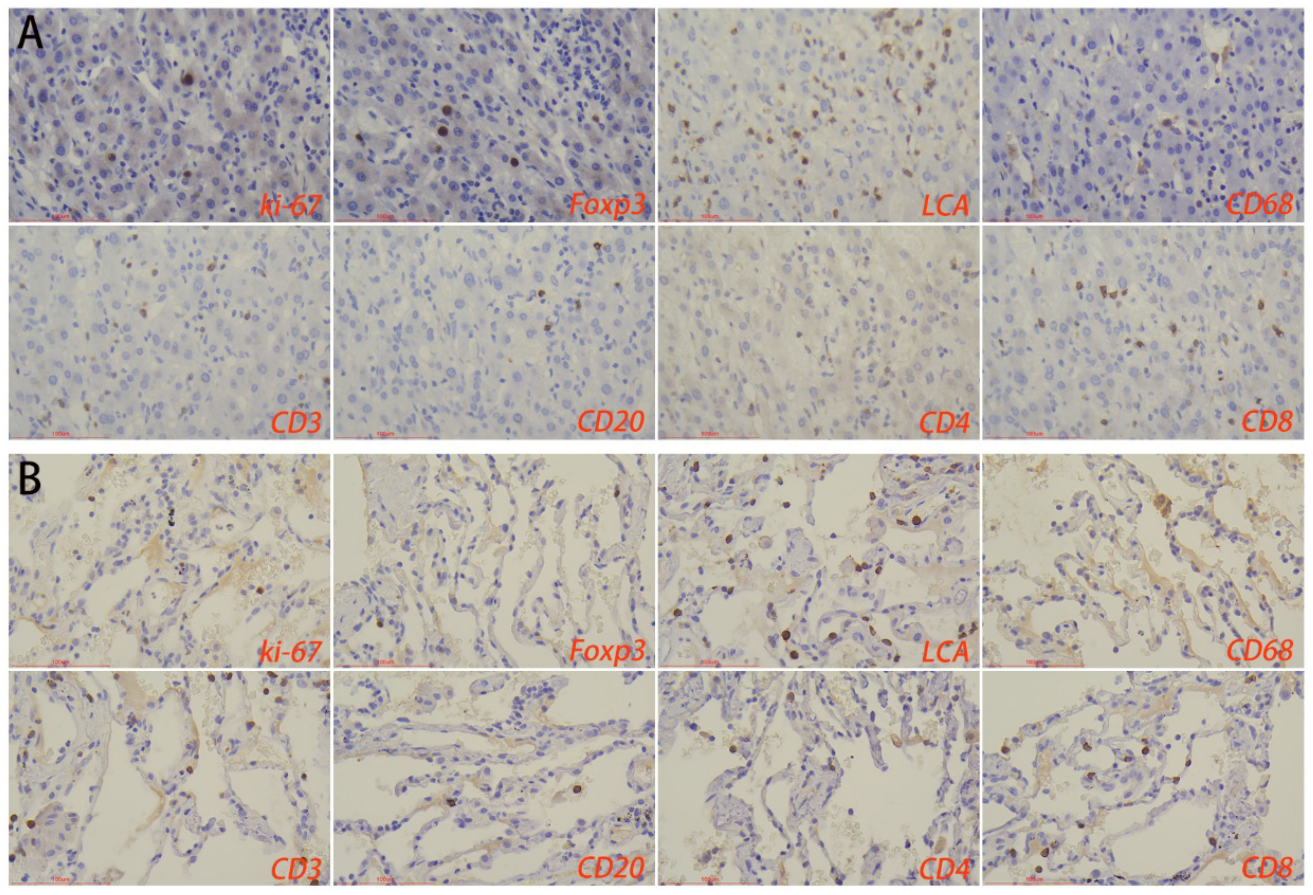
Immune microenvironment of normal liver tissues surrounding the lesion in **(A)** hepatic tuberculosis and **(B)** pulmonary tuberculosis. Immunohistochemical staining was used to label proliferation rate (ki-67^+^), immunosuppressive cells (Foxp3^+^), lymphocytes (LCA^+^), macrophages (CD68^+^), T-cells (CD3^+^), B-cells (CD20^+^), CD4^+^ T-cells, and CD8^+^ T-cells. Image magnification: immunohistochemical staining (200×).

Immunohistochemical results showed that the number of macrophages and foxp3^+^ cells in the normal liver tissue surrounding the lesions in the HTB group was significantly higher than that in the PTB group. There was no statistical difference in the number of lymphocytes, T-cells, B-cells, CD4^+^ T-cells, CD8^+^ T-cells, as well as the proliferation rate between the two groups (**Figure 6**; **Table 2**).

### 3.6 Drug resistance characteristics

Among the 32 MTB-positive specimens in the HTB group, 9.38% (n = 3) were sensitive to all four drugs, and 90.63% (n = 29) were resistant to any one drug. The overall monoresistance rate was 37.50% (n = 12), with the most resistant being INH (31.55%, n = 9); the multidrug resistance rate was 21.88% (n = 7), with the most resistant being RFP+INH+STR+EMB (12.50%, n = 4); the polyresistance rate was 31.25% (n = 10), with the most resistant being RFP+EMB (9.38%, n = 3).

Among the 32 MTB-positive specimens in the PTB group, 6.80% (2 cases) were sensitive to all four drugs, and 95.63% (n = 31) were resistant to any one drug. The monoresistance rate was 42.72% (n = 14), with the most resistant being INH (31.55%, n = 9); the multidrug resistance rate was 18.75% (n = 6), with the most resistant being RFP+INH+STR (9.38%, n = 3); the polyresistance rate was 31.25% (n = 10), with the most resistant being INH+STR (12.50%, n = 4). Overall, there was no statistical difference in the drug resistance characteristics of the patients between the two groups.

## 4 Discussion

PTB and HTB are two different types of tuberculosis, both of which are caused by MTB infection, but there are differences in epidemiological attributes, pathogenesis, and pathological characteristics (Jagdale et al., 2024). PTB usually occurs in patients with weakened immunity and is caused by inhaling droplets that contain MTB, leading to lung infections and respiratory symptoms such as cough, sputum, hemoptysis, chest pain, and fever. In severe cases, PTB may be life-threatening. HTB is a rare but serious type of tuberculosis, usually caused by the spread of tuberculosis bacillus to the liver through the blood circulation or lymphatic system (Li et al., 2021). In this study, 34.38% of the HTB patients had an explicit history of tuberculosis, and many patients may have had their lung lesions cleared at the time of diagnosis. We also found that HTB usually involves a single lesion, mainly occurring in the right lobe of the liver. The average volume of the lesion is smaller than that for PTB, which could be due to the solid structure of liver tissue. HTB sometimes manifests as symptoms of tuberculosis poisoning such as fatigue, night sweats, afternoon fever, bloating, and loss of appetite. In severe cases, it may cause biliary tract infections, jaundice, ascites, and hepatosplenomegaly, leading to irreversible liver damage (Maguire et al., 2020).

Enhanced CT is the most effective imaging method, characterized by circular delayed enhancement in the arterial, venous, and delayed phases. However, many case reports have shown that sometimes, it is difficult to distinguish between liver malignant tumors and HTB using enhanced CT (Ayadi et al., 2023; Koh and Leow, 2023; Si et al., 2023). Therefore, pathology plays a crucial role in the diagnosis and differential diagnosis of HTB.

Pathology is the gold standard for disease diagnosis. The typical pathological manifestation of tuberculosis is chronic granulomatous inflammation accompanied by caseous necrosis (Tsenova and Singhal, 2020). Among the pathological features of HTB, the frequency of granuloma and caseous necrosis was the most significant. Interestingly, this study shows that HTB has unique pathological features, with a significantly higher frequency of granulomas. Moreover, the share of granulomas in the entire lesion area was significantly bigger compared to pulmonary tuberculosis. The findings from this research suggest that the unique pathological features of hepatic tuberculosis could be mainly attributed to the immune microenvironment. There is a large number of macrophages in the liver tissue. After infection, MTB can immediately activate the body’s immune system, thereby releasing cytokines such as interleukin. However, long-term infection will continuously cause the patient’s physical fitness to deteriorate, having significantly reduced their immunity. The immunohistochemical results show that the number of macrophages in the normal tissue surrounding the tuberculosis focus is significantly higher than that in the lung tissue. MTB invades the liver and can be easily swallowed by macrophages. However, the liver has a higher inhibitory immune system compared to the lung and other organs (Chaudhry et al., 2019; Zheng and Tian, 2019). This study shows that the number of foxp3^+^ cells in the tuberculosis lesions and surrounding normal tissues of the HTB group is significantly higher than in the PTB group. Exaggerated immunity can cause caseous or inflammatory necrosis, while suppressed immunity can induce the formation of granulomatous lesions. In addition, structural characteristics may also be vital influencing factors, considering that the liver tissue is relatively denser, with smaller and fewer cavities. As a result, it’s relatively more difficult for the MTB to spread and the lesion to expand.

Although there are differences in pathological characteristics between PTB and HTB, both are caused by MTB infection and should be treated using standard anti-tuberculosis therapy (Goossens et al., 2020). Currently, isoniazid, rifampicin, and pyrazinamide are the anti-tuberculosis drugs with the strongest bactericidal activity, followed by streptomycin, ethambutol, ethioninib, and cycloserine. During the treatment of HTB, drug combinations should be selected based on the condition, body tolerance, and adverse reactions (Khawbung et al., 2021). In addition, healthy nutrition, along with protein supplements and vitamins are essential for supporting the treatment and improving the patient’s immune system and basic state (Kreutzfeldt et al., 2022). Most patients can be cured after the anti-tuberculosis treatment, but in cases of a too-large tuberculoma, liver abscess, or tuberculous cholangitis, the tuberculous focus should be removed on time. Regardless of whether it’s done before or after surgery, anti-tuberculosis treatment should be in accordance to the course of treatment (Malewadkar et al., 2021; Boldig et al., 2023). Understanding the drug resistance characteristics of HTB can aid precise and preventive treatment, considering that liver puncture carries potential risks. We compared the drug resistance characteristics of HTB and PTB and found no significant difference between the two groups. This indicates that the anti-tuberculosis treatment for hepatic tuberculosis can be carried out based on the treatment principles of PTB before biopsy. If conditions permit, a timely puncture biopsy should be performed to clarify the diagnosis and screen for sensitive drugs.

In conclusion, we found that there are substantial differences in the pathological characteristics and immune microenvironment between HTB and PTB. However, a significantly higher frequency of granuloma was noted in HTB compared to PTB. Similarly, the overall share of granulomas in the lesion area was also significantly larger. The immune microenvironment and structural characteristics may be important influencing factors. Our study provides important data support for the diagnosis and treatment of hepatic tuberculosis.

## Data Availability

The raw data supporting the conclusions of this article will be made available by the authors, without undue reservation.
